# Biometrics, e-Identity, and the Balance between Security and Privacy: Case Study of the Passenger Name Record (PNR) System

**DOI:** 10.1100/tsw.2011.48

**Published:** 2011-03-01

**Authors:** G. Nouskalis

**Affiliations:** Faculty of Law, Aristotle University, Thessaloniki, Greece

**Keywords:** biometrics, identity, Panoptikon, passenger, privacy, security

## Abstract

The implementation of biometrics entails either the establishment of an identity or tracing a person's identity. Biometric passport data (e.g., irises, fingers, faces) can be used in order to verify a passenger's identity. The proposed Passenger Name Record (PNR) system contains all the information necessary to enable reservations to be processed and controlled by the booking and participating air carriers for each journey booked by or on behalf of any person. PNR data are related to travel movements, usually flights, and include passport data, name, address, telephone numbers, travel agent, credit card number, history of changes in the flight schedule, seat preferences, and other information. In the aftermath of the September 11 attacks, a new emergency political-law status of society was established: the continuous state of “war” against the so-called unlawful combatants of the “enemy”. Officially, the enemy is the terrorists, but the victims of the privacy invasions caused by the above new form of data processing are the civilians. The data processing based on biometrics is covered both by Directive 95/46 EC and Article 8 of the Convention on the Protection of Human Rights and Fundamental Freedoms (now the European Convention on Human Rights, “ECHR”). According to Article 2, Paragraph a of the above Directive, personal data shall mean any information relating to an identified or identifiable natural person; an identifiable person is one who can be identified, directly or indirectly, in particular by reference to an identification number or to one or more factors specific to his/her physical, physiological, mental, economic, cultural, or social identity.

## INTRODUCTION

The implementation of biometrics entails either the establishment of an identity or tracing a person's identity. Biometric passport data (e.g., irises, fingers, faces) can be used in order to verify a passenger's identity. The published proposal of the European Commission for a Framework Decision on the use of

Passenger Name Record (PNR) data for law enforcement purposes, especially combating terrorism, raises security and privacy issues, which become more complicated due to the use of the above e-Passports[1].

The proposed PNR system contains all the information necessary to enable reservations to be processed and controlled by the booking and participating air carriers for each journey booked by or on behalf of any person. PNR data are related to travel movements, usually flights, and include the passport data, name, address, telephone numbers, travel agent, credit card number, history of changes in the flight schedule, seat preferences, and other information. The collection and analysis of PNR data allows the law enforcement authorities to identify high-risk persons and to take appropriate measures[2].

In the aftermath of the September 11 attacks, a new emergency political-law status of society was established: the continuous state of "war" against the so-called unlawful combatants of the "enemy". Officially, the enemy is the terrorists, but the victims of the privacy invasions caused by the above new form of data processing are the civilians. The problem is that some measures against terrorism, for example, an excessive data-processing system such as PNR, may seem reasonable in a situation of war, but they would never be acceptable in a time of peace. However, there is a tension between addressing terrorism as a crime and addressing it as a war.

The combination of the above PNR data and the system based on biometric, i.e., fingerprint or iris, recognition in passports provokes both new challenges and thinking about the balance between security and privacy. The condition for giving visa permission and the asylum policies are also relative matters. This paper attempts to clarify the main aspects of this subject and to bring into question the compatibility of the above biometric PNR database with the proportionality principle, which is fundamental in the processing of personal data in accordance with Directive 95/46 EC of the Convention on the Protection of Human Rights and Fundamental Freedoms (now the European Convention on Human Rights, "ECHR").

## THE LEGAL FRAMEWORK

The data processing based on biometrics is covered both by Directive 95/46 EC (hence "the Dir.") and Article 8 of the ECHR. According to Article 2, Paragraph a of the above Dir., personal data shall mean any information relating to an identified or identifiable natural person; an identifiable person is one who can be identified, directly or indirectly, in particular by reference to an identification number or to one or more factors specific to his/her physical, physiological, mental, economic, cultural, or social identity. In accordance with Article 8 of the ECHR, everyone has the right to respect for his private and family life, his home, and his correspondence. There shall be no interference by a public authority with the exercise of this right except such as is in accordance with the law, and as necessary in a democratic society in the interests of national security, public safety, or the economic well-being of the country, for the prevention of disorder or crime, for the protection of health or morals, or for the protection of the rights and freedoms of others. Respect for private life also consists of the right to establish professional or business relationships[3]. It is certain that public information also falls within the scope of private life, where it is systematically collected and processed in files held by the authorities. The ECHR has emphasized the correspondence of this broad interpretation with that of the Council of Europe's Convention of January 28, 1981 for the Protection of Individuals with regard to Automatic Processing of Personal Data; such personal data being defined in Article 2 as "any information relating to an identified or identifiable individual."

The provisions of the above piece of legislation constitute a concrete framework based on the following structure: The rule is that the processing is lawful when the data are processed fairly and in an adequate, relevant, and not excessive way in accordance with Article 5 of the Dir. Although a binding international agreement between the EU and the U.S. on privacy and data protection, in the context of the exchange of information for law-enforcement purposes, remains of the utmost importance, the EU seems to realize the necessity of a core or "privacy island" in the middle of the processing "ocean".

The above-proposed Framework Decision provides for the transfer or the making available by air carriers of PNR data of passengers of international flights to the Member States, for the purpose of preventing, detecting, investigating, and prosecuting "terrorist offenses or serious crime[4]." In accordance with Article 5 of the Dir., personal data should be collected for specified, explicit, and legitimate purposes, and not further processed in a way incompatible with those purposes. Further processing of data for historical, statistical, or scientific purposes shall not be considered as incompatible provided that Member States provide appropriate safeguards. In the EU data protection legal framework, the before-mentioned provisions generate the purpose specification principle. The purposes for which data are collected should be specified not later than at the time of data collection and the use of the data should be limited to the accomplishment of those purposes. The breach of that principle constitutes an unlawful processing of personal data.

In the explanatory report of the above proposal, it is mentioned that the scope of the proposal is limited to those elements that require a harmonized EU approach[5]. However, there is not a certain limitation about the extent of the collected data of so many people who are not officially either suspect or accused of any crime. Proportionality is often raised in general terms, without further explanation. The most critical question that relatively arises is the meaning of the proportionality principle and which factors are taken into account.

The principle of proportionality is a very important factor in the legal review of biometric systems. The question that arises is related to the specific criteria and factors used for evaluating the proportionality of processing biometric information. The application of the proportionality principle requires a certain duration of processing and a limited area of felonies that can be investigated through the collection of PNR data. According to the above proposal, the data are to be kept for 5 years, which constitutes a rather disproportionate invasion of privacy in order to fight uncertain threats if somebody takes into consideration that these data can be used for other purposes beyond fighting terrorism or serious criminality. It should also be noted that the general invocation of terrorism or serious crimes does not fulfill the requirement of purpose specification. There should be further clarification of the reason for the processing of the data.

## THE NEW LEGAL NOTION OF PRIVACY IN THE POSTMODERN CONTEXT OF A CONTINUOUS FIGHT AGAINST TERRORISM AND SERIOUS OR ORGANIZED CRIME

Today, the above processing is not the ultima ratio of data protection law based on fighting against criminals, however, a proper process through which a structure of security can be ensured. That acknowledgment implies some thoughts about justification of the related impact on privacy.

The enhancing of security in order to assist criminal law enforcement agencies through the above PNR system constitutes a new, postmodern Panoptikon[6], as it is described sociologically in terms found in the work of Michel Foucault[7]. The majority of the people can be considered as suspects of crime through the collection, storage, and processing of the above PNR data used by law enforcement agencies based on the invalidation of the presumption of innocence, in a permanent state of exception. In this context, the majority can be accounted as internal and unlawful combatants of the enemy in a war between a State and its citizens[8]. Thus, a legal framework based on the exceptional processing of personal data cannot adjust to the new rule of collection, storage, and processing in order to fight terrorism and serious crime.

## CONCLUSIONS

The published proposal of the European Commission for a Framework Decision on the use of PNR data for law enforcement purposes raises security and privacy issues in a postmodern era, which could entail a wide interpretation of a justification of the above data processing based on the continuous fight against terrorism and serious crime. The provisions of the EU data-protection law based on the exceptional processing of data cannot apply in the new environment in which the majority of the people are considered as suspects of crime.
